# Surgical Management of The Endomyocardial Fibrosis of Right Ventricle Mimicking Tumor with Recurrent Pulmonary Embolism

**DOI:** 10.21470/1678-9741-2020-0601e

**Published:** 2022

**Authors:** Reda Bzikha, Sébastien Queron

**Affiliations:** 1 Université of Paris Descartes, Sorbonne Paris-Cité, Paris, France; 2 Department of Cardiovascular Surgery, AP-HP, European Georges Pompidou Hospital, Paris, France

**Keywords:** Pulmonary Embolism, Endomyocardial Fibrosis, Magnetic Resonance Imaging, Heart, Thrombosis, Early Diagnosis.

## Abstract

Endomyocardial fibrosis is a neglected tropical disease that leads to restrictive cardiomyopathy. Its etiopathogenis is unclear and involves the progression of 3 stages of the disease.

Compared with echocardiography, cardiac magnetic resonance imaging shows better apical visualization of obliteration and thrombus and provides an early diagnosis.

However, there is no specific drug therapy, although surgery can increase survival. Therefore, surgical resection of the fibrous and thickened endocardium is recommended for symptomatic patients.

The risk of mortality increases as the ratio of endocardial fibrous tissue per body surface rises.

The aim of this manuscript is to describe the surgical management of the right-sided endomyocardial fibrosis mimicking tumor with recurrent pulmonary embolism.

## INTRODUCTION AND SURGICAL TECHNIQUE

**Fig. 1:**
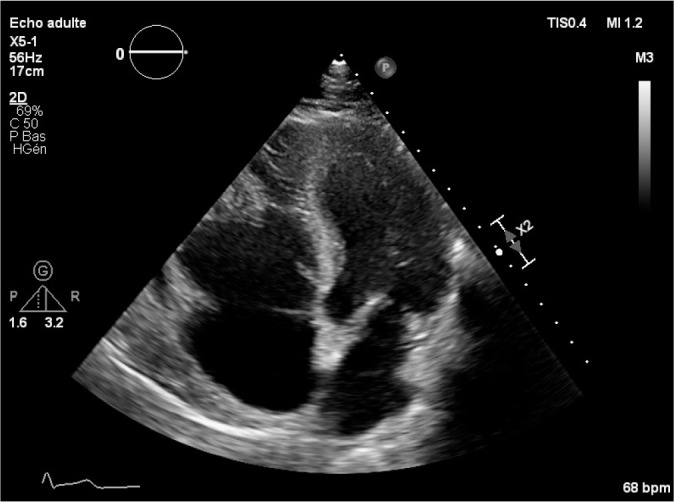
Obliteration of the right ventricle in the apical region, with polylobed mass in the right ventricular outflow tract. Note the thickened endocardium and the tricuspid annulus dilatation.

Our patient is a 44-year-old caucasian man who had a two-year history of recurrent pulmonary embolism. Its transesophageal echocardiography revealed the presence of an isoechoic, heterogeneous, and polylobed mass located in the infundibulum of the right ventricle, which measured 33 × 30 mm. this mass was associated with severe tricuspid regurgitation and normal pulmonary pressure. The systolic and the longitudinal right ventricle functions were normal (Figure 1).

**Fig. 2:**
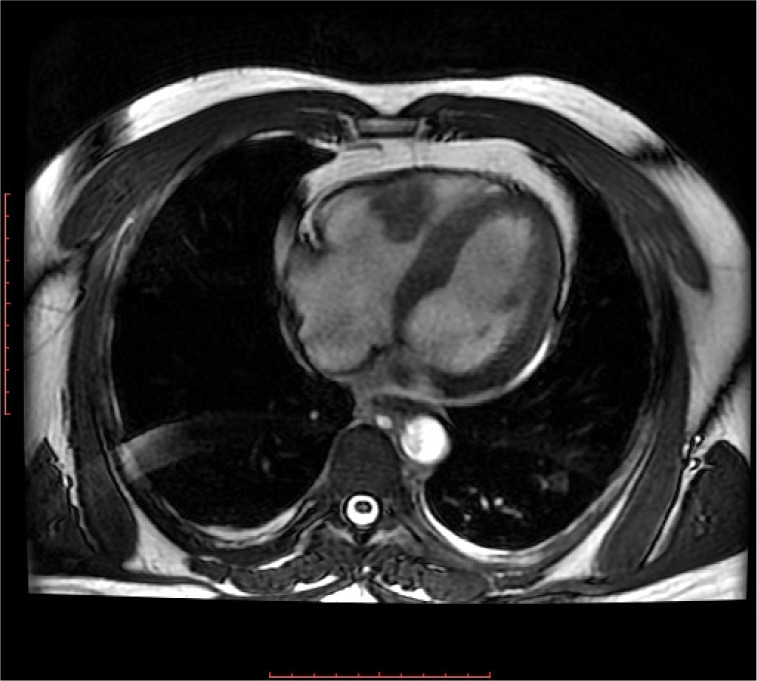
Steady-state free precession four-chamber cine view of cardiac magnetic resonance imaging demonstrating an apical obliteration of the right ventricle with a mass-like appearance protruding into the cavity from the right ventricular free wall. Note the pericardial effusion.

Gadolinium-enhanced cardiac magnetic resonance imaging confirmed the heterogeneous enhancement of this mass, which extended into right ventricular outflow tract (Figure 2).

We scheduled an emergency surgical removal of the mass in order to reduce the risk of embolism, as well as to rule out the tumor. The procedure was performed under cardiopulmonary bypass using aorto-bicaval cannulation. After aortic cross-clamping, the myocardial protection was achieved using anterograde crystalloid cardioplegia. The left ventricular vent was placed through the right upper vein.

Next, the right ventricle approach was made via an arciform transversal opening of the right atrium. At the inspection, we found a retraction of the right-sided chambers and a lack of mobility of the anterior and the posterior leaflets of the tricuspid valve. This retraction made the examination difficult of the ventricular cavity. In the view of this finding, the tricuspid valve considered being not repairable, and we decided to replace it with a bioprosthesis valve after the excision of the mass. As the resection of this valve was made, we found a mass in the right ventricle cavity that is subendocardial, indurate, smooth, yellowish and lobulated, invading the anterior papillary muscle, which caused the retraction of both the anterior and posterior leaflets. We should affirm that the posterior limit of this pathological tissue was invisible and difficult to be visualized.

**Fig. 3:**
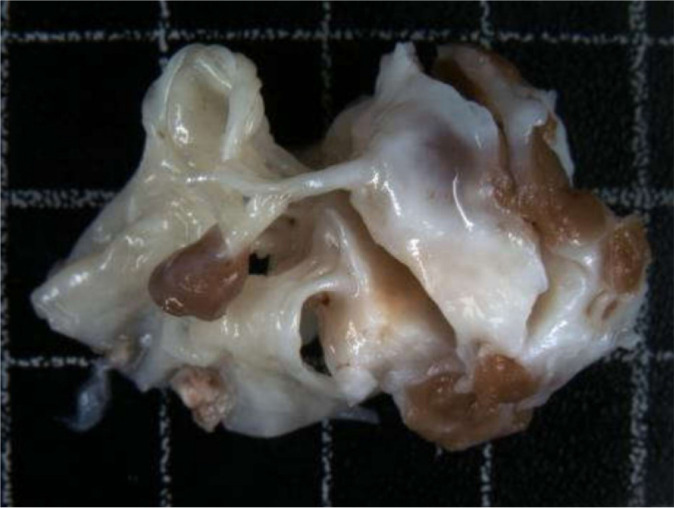
A very thickened and fibrotic endocardium and a tricuspid valve leaflet of heterogeneous thickness, with thickened and retracted chordae tendineae.

**Fig. 4:**
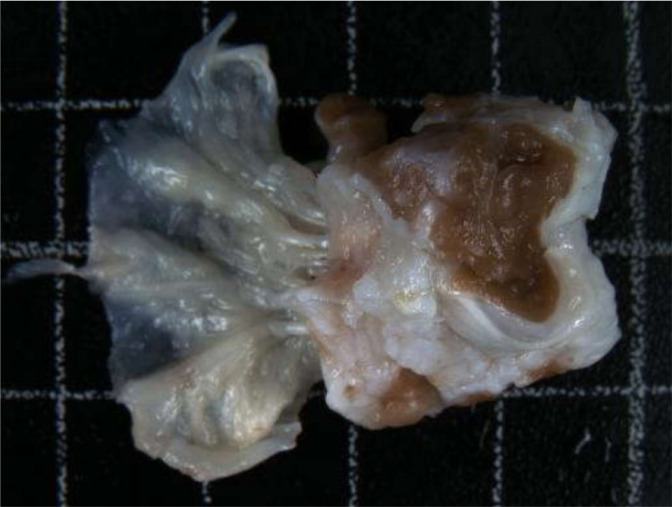
A thickened tricuspid valve leaflet of heterogeneous thickness, with thickened and retracted chordae tendineae.

And afterwards the excision of the anterior papillary muscle showed that it was invaded by a yellow tissue instead of the normal muscle and we revealed that the mass was subendocardial that extended to the apex of the heart (Figures 3 and 4).

However, a total resection of all the fibrotic tissue was difficult to be performed, as it was deeply invading the ventricular wall in its thickness. Yet, as the right ventricular cavity regained normal volume without any obstruction, we decided to stop the resection.

Thereafter, we performed a replacement of the tricuspid valve with a Carpentier-Edwards Perimount Magna Mitral Ease no. 31 bioprosthesis. After deairing maneuvers, we released the aortic cross-clamp. Then, spontaneous cardiac contractility was resumed, and the patient was successfully weaned from cardiopulmonary bypass. However, a complete atrioventricular block requiring a pacemaker placement, which was done percutaneously on the 10^th^ day, otherwise the follow-up was uneventful.

The histopathological examination confirmed endomyocardial fibrosis. The follow-up transesophageal echocardiography showed normal systolic and longitudinal right ventricle functions, and the bioprosthesis valve was considered competent, without any functional deficits.

## DISCUSSION

The endomyocardial fibrosis is a neglected tropical disease that leads to restrictive cardiomyopathy^[1]^. Its etiopathogenesis is unclear, but some authors have shown that there is an association between hypereosinophilic syndrome, cardiotropic infectious agents and endomyocardial syndrome^[1]^. This disease involves the progression of three stages: an active inflammatory phase known as acute phase, a transitional phase that is rare to be identified and a chronic fibrotic phase^[3]^.

The chronic phase is characterized by the rigidity and the thickening of the endocardium, especially in the inflow tract and the apex of one or both ventricles, but also the atria can be affected. In addition, the fibrosis of the atrioventricular valves and subvalvular apparatus lead to valve regurgitation.

This lesion causes restrictive cardiomyopathy that cause cardiac failure, thrombosis and calcification^[1]^. Also, it may lead to cardiac conduction disturbances^[2]^.

Concerning imaging investigation, the echocardiography is used to diagnose and to evaluate the severity of endomyocardial fibrosis. Besides, the ventricular thrombi can determine ventricular obliteration, the endocardial thickening can be seen as hyperechoic tissue. As well, the fibrosis of the ventricular apex described as Merlon sign, which is characterized as hypercontractile basal ventricle and hypokinetic/akinetic apex. And as square-root sign on M-mode of the septal and the posterior wall of the mid ventricle during diastole^[1]^.

Though, the cardiac magnetic resonance imaging provides better apical visualization of the obliteration and the thrombus as well as an early diagnosis of this disease.

The endomyocardial biopsy remains the sole way to confirm the diagnosis of this disease^[1]^.

Concerning the treatment of the endomyocardial fibrosis, we should declare that there is no specific drug therapy. However, surgery can increase survival^[2]^. Therefore, the surgical resection of the fibrous and the thickened endocardium is recommended for symptomatic patients^[4]^.

Dubost is one of the first cardiac surgeons to describe the surgical technique for endomyocardial fibrosis, he showed that this disease occurs in the filling chamber of one or both ventricles, in the papillary muscles, in the chordae tendineae and in the valve leaflets. He also showed that it never involves the ventricular outflow tract neither the atrioventricular annulus nor the aortic orifice^[5]^.

For Dubost, the surgical procedure is based on the decortication and the resection of the subvalvular apparatus in order to find the cleavage plane between fibrous tissue and the underlying myocardium. Starting the incision from the junction of valvular annulus and the fibrotic tissues then pursuing the dissection towards the ventricular apex where the adhesions are usually dense. Dubost also demonstrated that the thickened endocardium has to be completely excised for the reason to improve the diastolic function of the ventricle^[5]^.

So, the pitfalls are to remain in contact with the fibrosis without cutting into the adjacent myocardium^[5]^, to start the resection of the fibrous tissue from the atrioventricular orifice to the apex and to perform the valve replacement as it is recommended, rather than valve repair as the disease process^[5]^.

Concerning the treatment of the right ventricular dysfunction, the Fontan and the bidirectional cardiopulmonary shunt are reported in so many cases of the endomyocardial fibrosis^[6]^.

The early postoperative mortality rate is around 20%. And the postoperative complications are marked by low cardiac output syndrome and by atrioventricular block^[2]^. Thus, the recurrence rate of endomyocardial fibrosis ranges between 6% and 18.8%, and the risk of mortality increases as the ratio of endocardial fibrous tissue per body surface rises^[7]^.

## CONCLUSION

The endomyocardial fibrosis is a tropical disease that leads to restrictive cardiomyopathy. There is no specific drug therapy, but surgery remains recommended for symptomatic patients, as it increases survival. However, there some surgical pitfalls to follow in order to obtain good results and to improve prognosis of the patients.

**Table:** 

Authors' roles & responsibilities	
RB	Substantial contributions to the conception or design of the work; or the acquisition, analysis or interpretation of data for the work; final approval of the version to be published
SQ	Substantial contributions to the conception or design of the work; or the acquisition, analysis; or interpretation of data for the work; final approval of the version to be published
